# Color Face Recognition Based on Steerable Pyramid Transform and Extreme Learning Machines

**DOI:** 10.1155/2014/628494

**Published:** 2014-01-16

**Authors:** Ayşegül Uçar

**Affiliations:** Mechatronics Engineering Department, Engineering Faculty, Firat University, 23119 Elazig, Turkey

## Abstract

This paper presents a novel color face recognition algorithm by means of fusing color and local information. The proposed algorithm fuses the multiple features derived from different color spaces. Multiorientation and multiscale information relating to the color face features are extracted by applying Steerable Pyramid Transform (SPT) to the local face regions. In this paper, the new three hybrid color spaces, *YSCr*, *Z* ^*n*^ *SCr*, and *B* ^*n*^ *SCr*, are firstly constructed using the *Cb* and *Cr* component images of the *Y* *Cb* *Cr* color space, the *S* color component of the *HSV* color spaces, and the *Z* ^*n*^ and *B* ^*n*^ color components of the normalized *XYZ* color space. Secondly, the color component face images are partitioned into the local patches. Thirdly, SPT is applied to local face regions and some statistical features are extracted. Fourthly, all features are fused according to decision fusion frame and the combinations of Extreme Learning Machines classifiers are applied to achieve color face recognition with fast and high correctness. The experiments show that the proposed Local Color Steerable Pyramid Transform (LCSPT) face recognition algorithm improves seriously face recognition performance by using the new color spaces compared to the conventional and some hybrid ones. Furthermore, it achieves faster recognition compared with state-of-the-art studies.

## 1. Introduction

Color information of face images is very important for face recognition [1]. In [1–3], it was demonstrated that facial color features could drastically improve recognition performance compared with gray based cues. The *RGB* color space consists of a combination of the red, green, and blue components of images. The other color spaces are derived from *RGB* color spaces by linear or nonlinear transformations. Many recent works about face recognition have used the different color spaces in order to improve the recognition performance [1–8].

Two normalized hybrid color space methods were developed in [5]. In [6], the conventional color spaces such as *HSV*, *RGB*, and *Y* *Cb* *Cr* were evaluated comparatively with respect to each other and with respect to gray space by using Principal Component Analysis (PCA). In [7], a question of what kind of color space is suitable for color face recognition was surveyed and a set of optimal coefficients to combine the *R*, *G*, and *B* color components by a discriminant criterion was found. In [8], a new hybrid color space combining the *RGB* and *YIQ* color spaces was proposed. The results revealed that the hybrid color space is more powerful than gray space and even more than *RGB* color space. Some authors generated a new color space as *R* *Cr* *Q* by taking the *R*, *Cr*, and *Q* color components of *RGB*, *Y* *Cb* *Cr*, and *YIQ* color spaces into consideration sequentially in [9]. In this approach, Gabor Transform, Local Binary Patterns (LBP), and Discrete Cosine Transform (DCT) were applied to the *R*, *Cr*, and *Q* chromatic component images, respectively. All information obtained from the three color component images was fused by weighed sum rule. In [10], a new Discriminative Color Features (DCF) method was presented on the *RGB* *r* color space obtained by means of subtraction of the primary *G* and *B* color component images from primary *R* component image. In [1], Canonical Correlation Analysis (CCA) was presented for face feature extraction and recognition. In [11], Gabor wavelet and LBP were individually applied to *R* *Cr* *Q* color space and normalized *ZRG* color space proposed in [5] and the outputs of each classifier were combined by the feature fusion and the decision fusion. Although there are many studies, to determine the best hybrid color space is still a challenging problem for face recognition.

This paper evaluates different hybrid color spaces for improving face recognition performance by proposing Local Color Steerable Pyramid Transform (LCSPT) algorithm. Steerable Pyramid Transform (SPT) is a linear multiscale, multiorientation image decomposition technique [12]. SPT aims to represent the original image at different resolutions. Thus, the face image is analyzed by enhancing and isolating image features. SPT was successfully applied on gray images for face recognition in [13].

In color face recognition, the novelties presented in this paper are three fold.

(1) Firstly, an effective color feature extraction algorithm that increases the performance of face recognition by using SPT is proposed. In the algorithm, the features relating to each color component of color face images are extracted by using SPT at different angles and different scales.

(2) Secondly, three novel hybrid color spaces are proposed.

(3) Thirdly, a group of classifiers is applied to the efficient feature set obtained by using SPT with respect to decision frame. In this study, the Extreme Learning Machines (ELMs) for Single Layer Feed-forward Neural Networks (SLFNNs) are developed as an efficient classification method in color face recognition area. SLFNNs have been widely used in face recognition due to their approximation capabilities for nonlinear mappings using input samples. The weights and biases parameters of SLFNNs are usually iteratively adjusted by gradient-based learning algorithms. The past studies in the field of face recognition show that they are generally very slow due to improper learning steps or may easily converge to local minima and they need a number of iterative learning steps in order to obtain better learning performance [14–18]. To get rid of these limitations of SLFNNs for color face recognition in this paper, ELM proposed by Huang et al. [14] is suggested and the combination of ELM and SPT are used. In ELM, the weights of hidden nodes and biases are randomly chosen and output weights are analytically determined. ELM reaches to a good generalization performance in an extremely fast period [18]. ELM has been successfully applied to face recognition area [19, 20]. Also ELM has not been applied together with SPT in the face recognition literature. In this study, ELM and SPT were applied to color face recognition the first time. Comparative and extensive experiments have been illustrated to present the effectiveness of a new algorithm on the color FERET database [21] and the AR database [22].

The rest of this paper is organized as follows. In Section 2, SPT is shortly introduced. The basic architecture of ELM classifier is presented in Section 3. In Section 4, the types of color spaces are introduced.  Section 5 describes the proposed LCSPT face recognition algorithm. In Section 6, the comparative experimental results are illustrated. The paper is concluded in Section 7.

## 2. Steerable Pyramid Transform

The Steerable Pyramid is a multiorientation, multiscale image decomposition method proposed by Freeman and Adelson as an alternative to wavelet transform [12]. In SPT, an image is decomposed into noncorrelated frequency subbands localized in different orientations at different scales. The transform has steerable orientation subbands and a tight frame referred to as self-inverting. Steerable function means that it can be represented as a linear combination of rotated versions of itself. Self-inverting transform means that the synthesis function is the same as the analytical function. The combination of these two properties results in that the subbands become invariant from translation and rotation.

**Figure 1:**
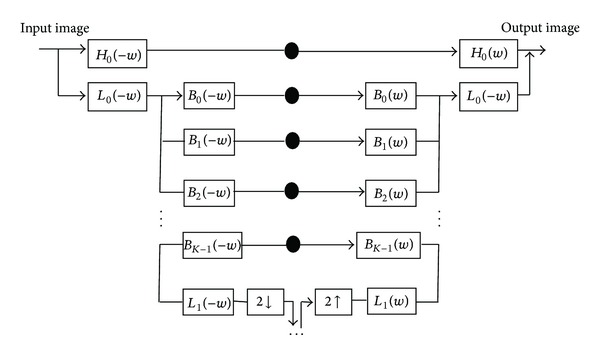
Block diagram of pyramid decomposition [12].

As shown in Figure 1, the input image is firstly decomposed into a highpass subband using a nonoriented highpass filter *H*_0_(*w*) and then into a lowpass subband using a narrow band lowpass filter *L*_0_(*w*). Afterwards this lowpass subband is decomposed into *K*-oriented portions using the bandpass filters *B*_*k*_(*w*) (*k* = 0,1,…, *K* − 1) and into a lowpass subband *L*_1_ [12]. The decomposition is done recursively by subsampling the lower lowpass subband. The small black boxes represent decomposed subband images. 2↓ and 2↑ indicate downsampling and upsampling by a multiplier of 2 along the rows and columns. Recursive steps extract different directional information at a given scale *J*.

The lowpass filters and highpass filters are defined in the Fourier domain by [12]

(1) $$\begin{array}{c} L_{0}\left(r,\theta \right)=L\left(\frac{r}{2},\theta \right),\\ H_{0}\left(r,\theta \right)=H\left(\frac{r}{2},\theta \right), \end{array}$$

where *r* and *θ* are the polar frequency coordinates. Consider

(2) $$\begin{array}{c} L\left(r,\theta \right)=\{\begin{array}{cc} 2\cos\left(\frac{\pi }{2}\log_{2}\left(\frac{4r}{\pi }\right)\right), & \frac{\pi }{4}<r<\frac{\pi }{2}\\ 2 & r\geq \frac{\pi }{4}\\ 0 & r\leq \frac{\pi }{2} \end{array}\\ B_{k}\left(r,\theta \right)=H\left(r\right)Q_{k}\left(\theta \right),\;k\in \left[0,K-1\right], \end{array}$$

where *B*_*k*_(*r*, *θ*) represents the *K*-directional bandpass filters used in the recursive steps with radial and angular parts, defined as

(3) $$\begin{array}{c} H\left(r\right)=\{\begin{array}{cc} \cos\left(\frac{\pi }{2}\log_{2}\left(\frac{2r}{\pi }\right)\right), & \frac{\pi }{4}<r<\frac{\pi }{2}\\ 1 & r\geq \frac{\pi }{2}\\ 0 & r\leq \frac{\pi }{4} \end{array}\\ Q_{k}\left(\theta \right)=\frac{\left(K-1\right)!}{\sqrt{K\left[2\left(K-1\right)\right]!}}{\left[2\cos\left(\theta -\frac{\pi k}{K}\right)\right]}^{K-1}. \end{array}$$

**Figure 2:**
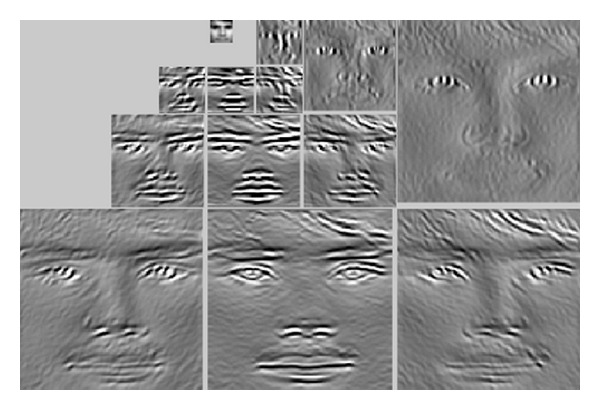
Steerable Pyramid Transform (*K* = 4 and *J* = 3) on *R* component of a cropped original color FERET image.

Figure 2 shows all filtered images at 3 scales (128 × 128, 64 × 64, and 32 × 32) and 4 orientation subbands (−*π*/4, 0, *π*/4, and *π*/2) on *R* component image of a cropped original FERET image. The SPT can locally detect the multiscale edges of facial images [13]. Detected features are noticeable by the first visual area of human visual cortex. In SPT, the lowest spatial-frequency subbands include distinctive edge information, whereas the higher spatial-frequency subbands contain finer edge information. The SPT coefficients consist of much redundant or irrelevant information. A suitable combination of these subbands can provide superior results. In [23], facial expression recognition was carried out by using only one subband.

## 3. Extreme Learning Machine

**Figure 3:**
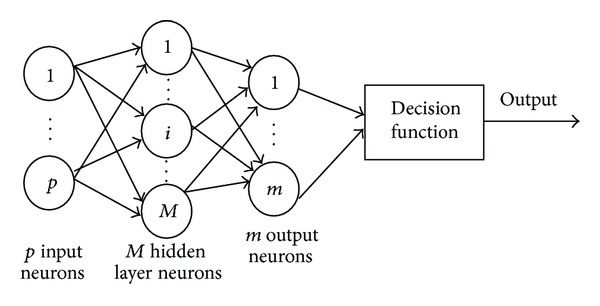
A simple ELM architecture.

The architecture of a simple conventional ELM proposed by Huang et al. [14–18] which is shown in Figure 3 is similar to SLFNNs with *M* hidden neurons and common activation functions.

Suppose we have a set of *N* arbitrary distinct samples (*x*_*i*_, *y*_*i*_) where *x*_*i*_ = [*x*_*i*1_,*x*_*i*2_,…,*x*_*ip*_]^*T*^ ∈ *R* ^*p*^ is a *p*-dimensional input vector and *y*_*i*_ = [*y*_*i*1_,*y*_*i*2_,…,*y*_*im*_]^*T*^ ∈ *R* ^*m*^ is an *m*-dimensional output vector. The input-output relation of a conventional ELM with *M* hidden nodes and activation function *g*(*x*) has the form

(4) $$\begin{array}{c} \sum_{i=1}^{M}\beta_{i}g\left(x_{k}\right)=\sum_{i=1}^{M}\beta_{i}g\left(w_{i}\cdot x_{k}+b_{i}\right)=o_{k},\;k=1,\ldots ,N, \end{array}$$

where *w*_*i*_ = [*w*_*i*1_,*w*_*i*2_,…,*w*_*ip*_]^*T*^ ∈ *R* ^*p*^ means the weights between the inputs nodes and an *i*th hidden node, *β*_*i*_ = [*β*_*i*1_,*β*_*i*2_,…,*β*_*im*_]^*T*^ ∈ *R* ^*m*^ means the weights between an *i*th hidden node and output nodes, and *b*_*i*_ means the threshold of an *i*th hidden node.

The conventional ELM in (4) can approximate *N* samples with zero error by satisfying

(5) $$\begin{array}{c} \sum_{k=1}^{N}||o_{k}-y_{k}||=0 \end{array}$$

or

(6) $$\begin{array}{c} \sum_{i=1}^{M}\beta_{i}g\left(w_{i}\cdot x_{k}+b_{i}\right)=y_{k},\;k\in \left\{1,2,\ldots ,N\right\}. \end{array}$$

The output of ELM can be written more compactly in matrix form as

(7) $$\begin{array}{c} G\beta =Y, \end{array}$$

where

(8) G(w1,…,wM,b1,…,bM,x1,…,xN) =[g(w1·x1+b1)⋯g(wM·x1+bM)⋮⋯⋮g(w1·xN+b1)⋯g(wM·xN+bM)]N×M,βi=[βi1,βi2,…,βim]TM×m,  Yi=[y1,y2,…,yN]TN×m.

In (8), *G* is the hidden layer output matrix of ELM and *g* is infinitely differentiable activation function; the number of hidden nodes is chosen as *M* ≪ *N*. Here, the ${\hat{w}}_{i},{\hat{b}}_{i},\hat{\beta }\;\;(i=1,\ldots ,M)$ parameters of conventional SLFNN are adjusted by solving the primal optimization problem:

(9) ||G(w^1,…,w^K,b^1,…,b^K)β^−Y|| =minwi,bi,β ||G(w1  ,…,wM,b1  ,…,bM)β−Y||.

The objective function for optimization problem in (9) is expressed as

(10) E=∑k=1N(∑i=1Mβig(wi·xk+bi)−yk)2.

The parameters are optimized by calculating the negative gradients of objective function in (10) with respect to *w*_*i*_, *b*_*i*_, *β*_*i*_. Consider

(11) wj=wj−1−τ∂E∂W.

The accuracy and learning speed of gradient based method particularly depend on the learning rate, *τ*. Small learning rate provides very slow convergence, whereas a larger learning rate exhibits the bad local minima effect. ELM uses minimum norm least-square solution to get rid of these limitations. The weight and bias values of ELM are randomly assigned unlike SLFNNs. Output weights of ELM are analytically determined through a generalized inverse operation of the hidden layer weight matrices, since the learning problem is converted into a simple linear system. So it is obtained extremely fast with better generalization performance than those of traditional SLFNN for hidden layers with infinitely differentiable activation functions. The final ELM achieves not only the smallest training error but also the smallest generalization error thanks to the obtained smallest norm of output weights similar to Support Vector Machines (SVM) [24].

For randomly fixed weights in the hidden nodes, the learning of ELM is equal to a least-square solution in (9). *G* is a nonsquare matrix for *M* ≪ *N*. ELM represented by the linear system in (7) gives a norm least-square solution as $\hat{\beta }=G^{*}Y={\left(G^{T}G\right)}^{-1}G^{T}Y$, where G* is the Moore-Penrose generalized inverse of matrix *G*. The smallest training error is achieved by using

(12) $$\begin{array}{c} ||G\hat{\beta }-Y||=||GG^{*}Y-Y||=\underset{\beta }{\min}||G\beta -Y||. \end{array}$$

The performance of ELMs having different activation functions has been presented for both regression and classification in [14–18]. In this paper, we are interested in the ELM classifiers with a sigmoid activation function.

## 4. Hybrid Color Spaces for Face Recognition

The hardware-oriented models including digital image processing commonly use *RGB* color space. Each pixel of a color image is represented in the hardware as binary values for the red, green, and blue color components. Different color spaces are used for different applications. Hence, the *RGB* color space can be converted to the desired color space by using the values in some formulation with respect to the application. The color components of *RGB* color space have largely correlation with each other. Hence the conventional color spaces such as *YIQ*, *Y* *Cb* *Cr*, L*a*b*, and *HSV* are more effective than original *RGB* color space at face recognition. In this paper, we investigated the color spaces in the literature and their hybrid color component combinations and their color component combinations for improving face recognition performance.

The *HSV* (hue, saturation value) color space is defined as follows [25]:

(13) H={θif  B≤G360−θif  B>GS=1−3(R+G+B)[min(R,G,B)],V=max(R,G,B),

where

(14) θ=cos−1{0.5[(R−G)+(R−B)][(R−G)2+(R−B)(G−B)]1/2}.

In this color space, hue (*H*) is a measure of the spectral composition of a color, saturation (*S*) shows the relative purity or the amount of white light mixed with a hue, and value (*V*) refers to the luminance of the image. This model is commonly used for face detection and skin detection [26–28].

The *Y* *Cb* *Cr* color space is given by [29]

(15) [YCbCr]=[0.2570.5040.098−0.148−0.2910.4390.439−0.368−0.071][RGB]+[16128128],

where *Cb* and *Cr* are chrominance components and *Y* is separating luminance component. This space is effective for skin color segmentation and face detection [26–28, 30].

The *YIQ* color space is computed by

(16) [YIQ]=[0.2990.58700.11400.5957−0.2745−0.32130.2115−0.5226−0.3111][RGB],

where *I* stands for in-phase and *Q* stands for “quadrature”, which is based on quadrature amplitude modulation. Consider

(17) [XYZ]=[0.6070.1740.2000.2990.5870.1140.0000.0661.116][RGB].

The L*a*b* color spaces are defined based on the *XYZ* tristimulus values by the following equations:

(18) L∗={116(YYn)1/3−16if  YYn>0.008856903.3(YYn)if  YYn≤0.008856a∗=500∗(f(XXn)−f(YYn)),b∗=200∗(f(YYn)−f(ZZn)),

where *X*_*n*_, *Y*_*n*_, and *Z*_*n*_ are the tristimulus values of the reference white point. Consider

(19) f(t)={t1/3if t>0.0088567.787∗t+16116if t≤0.008856.

The L*a*b* color space corresponds to brightness ranging from black (0) to white (100). a* component corresponds to the measurement of redness (positive values) or greenness (negative values). b* component corresponds to the measurement of yellowness (positive values) or blueness (negative values). This color space was effectively used for color face expression recognition in [31].

The normalized *XYZ* and *RGB* color spaces are obtained by using the across-color-component normalization technique in [4]. In this paper, the normalized *XYZ* and *RGB* color spaces are names as *XYZ*-*n* and *RGB*-*n*, respectively. The normalized color components, *X*, *Y*, *Z*, *R*, *G*, and *B* are named as *X* ^*n*^, *Y* ^*n*^, *Z* ^*n*^, *R* ^*n*^, *G* ^*n*^, and *B* ^*n*^, respectively. *XYZ*-*n* and *RGB*-*n* color spaces are defined as

(20) [XnYnZn]=[0.60700.17400.2000−0.09010.3631−0.2730−0.4600−0.19860.6586][RGB],[RnGnBn]=[100−0.57740.7887−0.2113−0.5774−0.21130.7887][RGB].

In [10], a simple effective model was generated by means of the subtraction of primary colors with respect to Ockham's razor principle. In this paper, the color space is named as RGB-r. The color components, *G* and *B*, are named as *Gr* and *Br*, respectively. Consider

(21) [RrGrBr]=[1001−1010−1][RGB],

Generally, the *HSV* and *Y* *Cb* *Cr* color spaces are the best color spaces used for skin detection and face detection [3, 26–28, 30, 31]. The *R* component images have fine face region [32]. The *Cr* and *Cb* component images contain partial face contour information [9]. The *S* and *V* components are the powerful component images for color face recognition [6, 29], whereas *RIQ* color space [8], *R* *Cr* *Q* color space [9], *RGB*-*r* color space [10], and *ZRG*-*n* color space consisting of the color components of *XYZ*-*n* and *RGB*-*n* color spaces [11] have been powerful color spaces for face recognition.

## 5. Feature Extraction for Color Face Recognition

This section details the novel color feature extraction and multiple feature combination methods for the proposed LCSPT face recognition algorithm. The algorithm incorporates features such as local spatial information and color information for improving face recognition performance. The color information is obtained by using novel hybrid color spaces derived from six conventional color spaces, *RGB*, *YIQ*, *Y* *Cb* *Cr*, *XYZ*, *HSV*, and L*a*b* and three hybrid color spaces, the *RGB*-*n* [5], *XYZ*-*n* [5], and the RGB-r [10]. The hybrid spaces in this paper are constructed by 3 components as in [33]. So the dominant features of each component image are merged.

**Figure 4:**
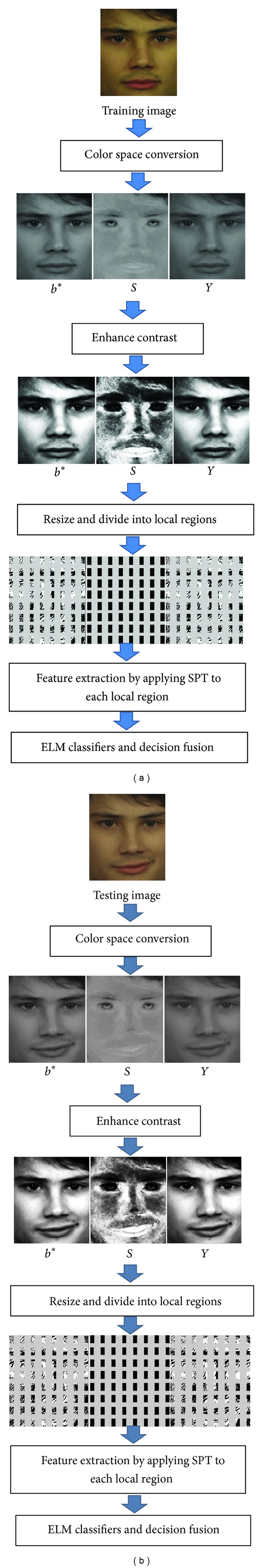
The block scheme of the proposed LCSPT algorithm.

Illustration of the proposed LCSPT face recognition algorithm is given in Figure 4. The algorithm is applied in five steps.

1. In this paper, new three color spaces, *YSCr*, *Z* ^*n*^ *SCr*, and *B* ^*n*^ *SCr* are constructed. The new hybrid color spaces consist of the *Cb* and *Cr* component images of the *Y* *Cb* *Cr* color space, the *S* color component of the *HSV* color spaces, and the *Z* ^*n*^ and *B* ^*n*^ color components of the normalized *XYZ* color space.
2. Each component contrast is enhanced and divided into local partitions by an efficient pixel number. An efficient pixel number is determined by taking the resolution of face image into consideration.
3. By applying SPT at a specific scale and a specific orientation to each local image portion, the statistical features such as mean, entropy, and variance of the local face images are extracted.
4. The group of ELM classifiers is employed to classify the statistical features relating to the color component images of each subband. A decision fusion system combines local decisions from each classifier in the group into a single decision. The combination is implemented by a product decision rule to generate a fused decision vector [34].

## 6. Experiments and Results

This section evaluates the effectiveness of the proposed LCSPT algorithm on possibly the most representative examples of color face recognition. We used the color FERET database [21] and the AR database for experiments [22]. The experiments cover a wide range of facial variability and moderately controlled capturing conditions: facial expression (AR and color FERET), illumination changes (AR and color FERET), aging (color FERET), and slight changes in pose (color FERET). Experiments performed were using a single sample per class for color FERET database as well as more than one sample per class for the AR database.

In color FERET database, we centered all face images with respect to their ground truth face coordinates in [21] cropped and scaled to 128 × 128 pixels resolution. We used the cropped AR face images in [35]. In particular, we applied SPT at 3 scales (128 × 128, 64 × 64, 32 × 32) and 4 orientation subbands (−*π*/4, 0, *π*/4, *π*/2) to the cropped images. The highpass subband is labeled as *HS* in all tables. In experiments, all subbands relating to only the first scale were used since the results of the first scale were better than the others. If the other scales were used for the face recognition, the recognition performance might increase too. However, the computation complexity will increase since the input space dimension will grow. We tried many efficient pixel numbers such as 4 × 4, 16 × 16, and 32 × 32. We obtained the best performances for both datasets by using an efficient pixel number of 8 × 8.

All the experiments are run on a personal notebook computer with 2.4-GHz Intel(R) Core(TM)2 Duo processor, 3 GB memory, and Windows 7 operation system. Comparative studies of ELM, SVM, k-Nearest-Neighbors (k-NN), and Feed-forward Neural Networks (FNNs) for the proposed LCSPT face recognition algorithm are carried out. In order to validate both the classification accuracy and the training and testing speeds of SVM, MATLAB interface LIBSVM 2.83 software implementing Sequential Minimal Optimization algorithm, decomposing the overall QP problem into QP subproblems, http://www.csie.ntu.edu.tw/~cjlin/libsvm, was used [36]. The values of kernel and regularization parameters were selected taken as 1/(2*σ* ^2^) = [2^4^, 2^3^, 2^2^,…, 2^−10^] and *C* = [2^12^, 2^11^, 2^10^,…, 2^−2^], respectively. 15 × 15 = 225 combinations of the parameters were generated. The best combination was searched [36, 37]. The parameters exhibiting the best 10-fold cross-validation accuracy on the training dataset were accepted as optimal ones as in [24, 36, 37]. 10-fold cross-validation divides the training set into 10 subsets of equal size, and sequentially one subset is tested using the classifier that was trained on the remaining 9 subsets.

ELM, having fast learning and testing speed, allows us to repeat the experiments several times. We changed hidden neuron number of ELM with sigmoid activation function to find the best number. We firstly took as 10 and then increased to the input sample size by the increasing step of 2. We searched ELM with the best correctness. ELM gives better results for large hidden neuron number [15]. On the other hand, an FNN can give good results for small hidden neuron number. The hidden neuron number of the FNNs with sigmoid function was determined in a range from 10 to the input sample size by steps of 2. The FNNs were trained using the conjugate gradient learning algorithm for 500 epochs. For k-NN, we changed the neighbor number from 1 to 5. We run every experiment for each classifier 10 times. Average results are reported in tables.

In addition, we compared the performance of our LCSPT face recognition algorithm with those of color Local Binary Decision (LBD) method in [38] and Local Color Vector Binary Patterns (LCVBP) method in [39]. We used the MATLAB source codes available in [38, 39]. For LBD, we used the local standard deviation filter with a window size of 7 × 5 pixels for a normalization window size of 80 × 90 with respect to the recommendations in [38]. For LCVBP, we rescaled to the size of 112 × 112 pixels and then divided into the local regions with the size of 18 × 21 pixels as in [39]. Our method performed the best recognition performance in all experiments.

We also tried the feature fusion frame for our algorithm. Decision fusion has slightly better performance with respect to the feature fusion in many subbands. The results relating to the feature fusion frame are not included in the tables in order not to corrupt the completeness of the paper. In addition, the feature fusion frame is used together with the dimension reduction techniques in general because it has a large number of features. This also means an additional computational cost. If the dimension reduction techniques are not used, the feature fusion frame requires large computational time.

### 6.1. Evaluation of Proposed LCSPT on AR Database

**Figure 5:**
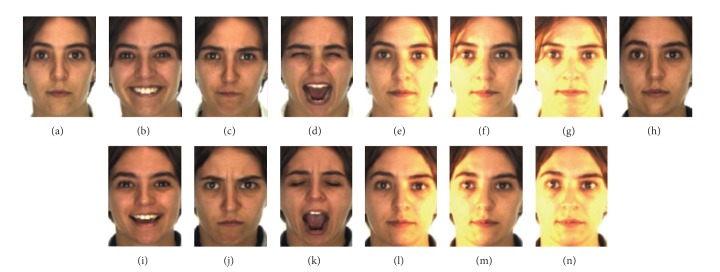
Sample images for one subject of the AR database. The images of (a) neutral expression, (b) smile, (c) anger, (d) scream, (e) left light on, (f) right light on, (g) all sides with light on, and the images of (h)–(n) under the second session.

The AR database [22] contains over 4,000 frontal view color face images of 126 subjects (76 men and 56 women). Each subject has up to 26 images taken in two sessions, separated by two weeks. Each session contains 13 images with different facial expressions, lighting conditions, and occlusions. The images of 100 subjects were used in our experiments [35].  Figure 5 shows the image samples relating to one person in the AR database used in our experiments. The images consist of neutral expression, smile, anger, scream, left light on, right light on, and all sides light on for both sessions under the same conditions. We planned two experiments on the AR database in order to study the robustness of the LCSPT face recognition algorithm. In the first AR experiment, we contained only the variation of facial expressions. We used four images (a)–(d) of session 1 for training and three images (i)–(k) of session 2 for testing. In the second AR experiment, we included both illumination variations and expression variations. We used seven images (a)–(g) of session 1 for training and seven images (h)–(n) of session 2 for testing. We did not consider the occluded face recognition in each session.

**Figure 6:**
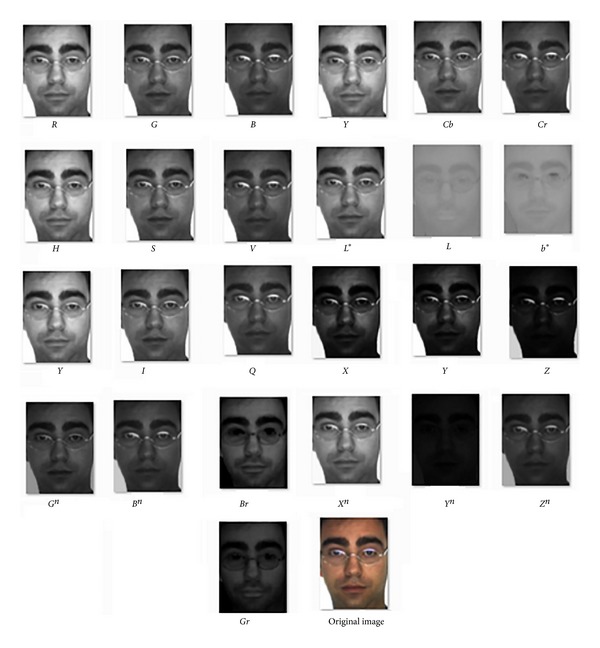
A subject of the AR database and its color component images.

Firstly, the performance of our algorithm on 24 color components (*R*, *G*, *B*, *H*, *S*, *V*, *Y*, *Cb*, *Cr*, *Y*, *I*, *Q*, *X*, *Z*, L*, a*, b*,*X* ^*n*^, *Y* ^*n*^, *Z* ^*n*^, *B* ^*n*^, *G* ^*n*^, *Gr*, and *Br*) of the 9 color spaces (*RGB*, *HSV*, *Y* *Cb* *Cr*, *YIQ*, *XYZ*, L*a*b*, *RGB*-*n* [5], *XYZ*-*n* [5], and *RGB*-*r* [10]) is constructed. The color component images of the evaluated color spaces are shown on an image belonging to the AR database as in Figure 6.  Table 1 presents our results on 24 color component images. The left side of Table 1 lists the results of AR experiment 1. These results indicate that the correctness at all subbands for *G*, *B*, *Y*, L*, *B* ^*n*^, *X* ^*n*^, and *Z* ^*n*^ color components are over 90%. However, the correctness of the other color components is below 90% in one or many subbands. Taking into consideration all the subbands for the *G*, *B*, *Y*, L*, *B* ^*n*^, *X* ^*n*^, and *Z* ^*n*^ color components, we observe the best results on the *Y* color component for HS subband, the L* color component for −*π*/4 subband, the *Z* ^*n*^ and *B* ^*n*^ color components for 0 subband, B for *π*/4 subband, and the *Y*, *Z* ^*n*^, and *X* ^*n*^ color components for *π*/2 subband. From these results, we conclude that the results of the *Y*, L*, *Z* ^*n*^, *B* ^*n*^, and *B* color components are better than the *G* color component and even the others for facial expression experiment. In the case of an experiment including the variation of facial expression, *Z* ^*n*^ and *B* ^*n*^ color components are the best in the color components with respect to the correctness in the their subbands since the fusion of the subbands with high correctness will provide a higher correctness.

The right side of Table 1 lists the results of AR experiment 2. These results indicate that the correctness at all subbands on only *B*, *Y*, *B* ^*n*^, *X* ^*n*^, and *Z* ^*n*^ color components is over 90%. Taking all the subbands for *B*, *Y*, *B* ^*n*^, *X* ^*n*^, and *Z* ^*n*^ color components into consideration, we observe the best results on *Y* and *X* ^*n*^ color components for *HS* subband, *Y* and *X* ^*n*^ color components for −*π*/4 subband, *Y* and *B* ^*n*^ color components for 0 subband, B for *π*/4 subband, and *Y*, *B* and *Z* ^*n*^ color components for *π*/2 subband. From these results, we conclude that *Y* color component is specifically better than the others for the experiment including illumination experiment. The results correlate with the literature [26–28, 30]. From all the results in Table 1, we infer that the color components, *Y*, *Z* ^*n*^, *B* ^*n*^, *X* ^*n*^, and *B* can be used to obtain an acceptable good performance in both experiments. Hence, we can give our priority to these components in an experiment including the variation of illumination.

We compared our new three hybrid color spaces to 9 color spaces of *RGB*, *HSV*, *Y* *Cb* *Cr*, *YIQ*, *XYZ*, L*a*b*, *RGB*-*n* [5], *XYZ*-*n* [5], and *RGB*-*r* [10] and the hybrid color space of *RIQ* [8], *R* *Cr* *Q* [9], and *ZRG*-*n* [5] and presented advantages with respect to the conventional RGB color space. In order to make our results clearer, we give only the best hybrid color spaces although we tried all combinations of 24 color components.  Table 2 shows the recognition correctness of all hybrid color spaces. Specifically, we obtained that the hybrid color spaces generated by using the *S* and *Cr* color components combined together with the *Y*, *B* ^*n*^, *X* ^*n*^, *B*, and *Z* ^*n*^ color components improve more effectively the face recognition performance. For two AR experiments, the results of the *YSCr*, *B* ^*n*^ *SCr*, and *Z* ^*n*^ *SCr* hybrid color spaces outperform those of the powerful conventional color spaces, the other color spaces and the individual color components such as *R*, *G*, *Y*, and *Z* ^*n*^. Moreover, we obtained the best results with the correctness of 99.45 in the *YSCr* hybrid color space. On the other hand, if one wants to have the higher correctness for each color component or each color space in Table 1 and Table 2, then all their subbands could be fused by the decision or feature fusion method [13, 20] in terms of more computational complexity.

In Table 3, we compared our results on the *YSCr* hybrid color space to LBD method in [38] and LCVBP method [39] in terms of the training time and the testing time and the recognition correctness. As can be seen from these results, our LCSPT-ELM face recognition algorithm is the best one in computing time especially. Moreover, the correctness of LCSPT-ELM outperforms the others.

**Figure 7:**
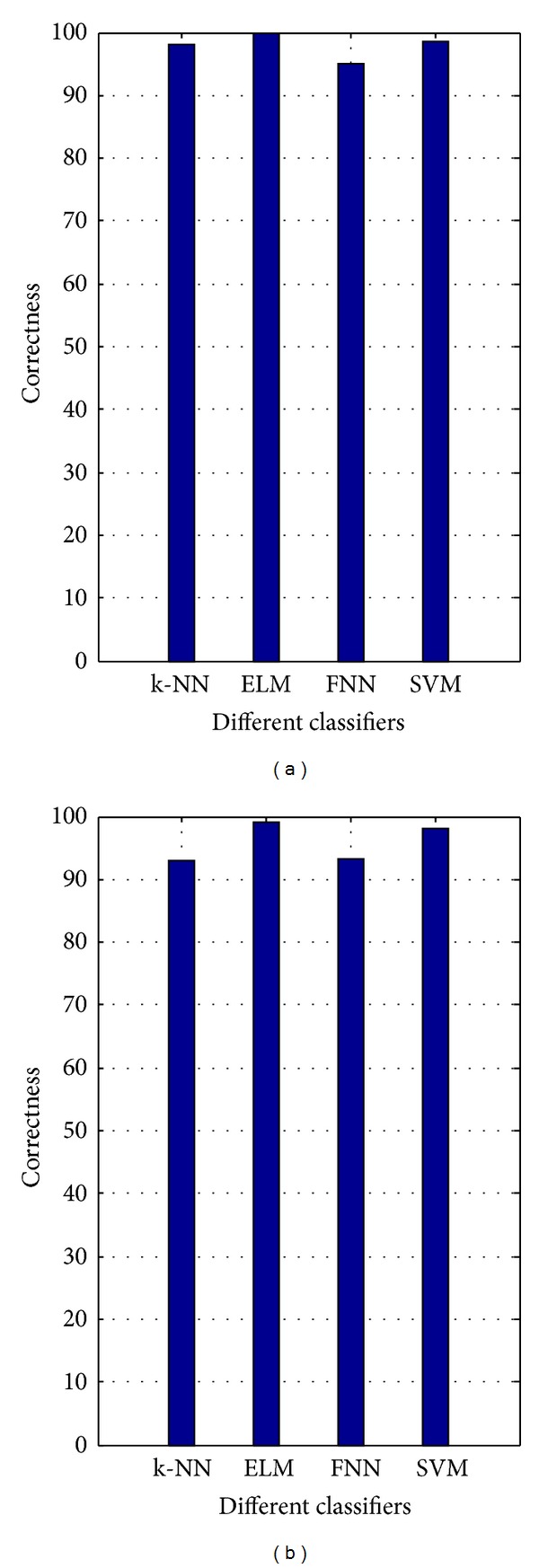
Performance comparison for k-NN, ELM, FNN, and SVM classifiers: AR database: (a) Experiment 1 and (b) Experiment 2.

We also compared the parameter adjusting time, the testing time, and correctness of SVM, k-NN, FNN, and ELM.  Figure 7 shows the correctness of all classifiers. ELM outperforms the others in terms of the correctness. In Table 4, the results on the *Y* color component image are given. FNN is the most time consuming method in with regard to the parameter adjusting time, but FNN has the shortest testing time due to the high compact network architecture [15]. The parameter adjusting time of k-NN is the fastest, however, its performance and testing time are worse than that of the ELM for both AR experiments. The advantage of ELM is obviously seen by taking both the correctness and training time into consideration. ELM runs around 6 times faster than SVM and 130 times faster than FNN. After the parameter adjusting process we obtained the optimum parameters for the AR experiments. The hidden neuron number of FNN and ELM, neighbor number of k-NN, and (*C*, 1/(2*σ* ^2^)) parameters of SVM are given in Table 4.

### 6.2. Evaluation of Proposed LCSP on Color FERET Database

**Figure 8:**
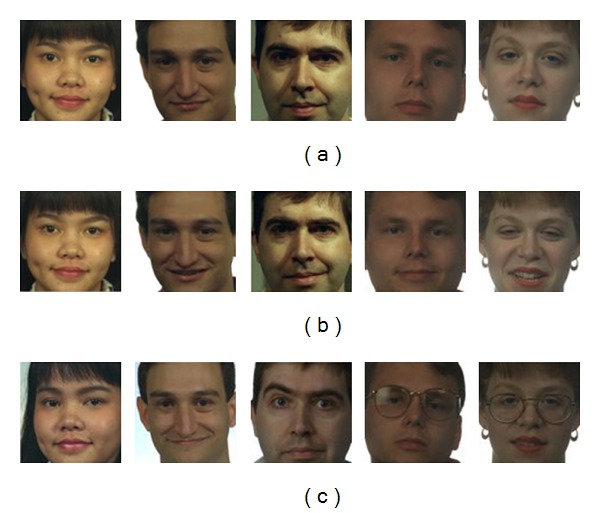
Some sample images of the subsets of (a) fa, (b) fb, and (c) dup1 of the color FERET database.

The FERET database consists of 11,388 color facial images obtained from 994 subjects being captured in the course of 15 sessions. The images have a resolution of 512 × 768 pixels. The database is very challenging due to significant appearance changes in the individual subjects in terms of aging, facial expressions, glasses, hair, moustache, nonuniform illumination variations, and slight changes in pose. The database is divided into five subsets: fa, fb, fc, dup1, and dup2. fa subset contains one frontal view per subject and in total 1196 subjects. We conducted single-sample-per-class face recognition experiments on the color FERET database [21]. The FERET evaluation methodology requires that the training processing be carried out using only the fa subset. We selected a subset of color face images of 204 persons. Their ground truth face coordinates are available in the color FERET database. We generated the training set by only single image portrait images consisting of 204 people from fa subset, whereas testing set was from the fb subset and the dup 1 subset.  Figure 8 shows some sample images relating to the subsets of fa, fb, and dup1 of the color FERET database used in our experiments.

The left side of Table 5 shows the results on the fb subset of color FERET database. We observe the best results on the *B*_*r*_ color component for *HS* subband, the *S* color component for −*π*/4 subband, the *Y*, *X*, and *I* color components for 0 subband, *Cb* color component for *π*/4 subband, and the *B* and *Z* ^*n*^ color components for *π*/2 subband. From these results, we conclude that *Br*, *S*, *Y*, *X*, *I*, *Cb*, *B*, and *Z* ^*n*^ color components are especially better than the others. The right side of Table 5 shows the results on the dup *I* subset of the color FERET database. As we can see from the right side of Table 5, the best color components are the *G*, *I*, *Y*, and *Z* ^*n*^. If we search the best color components in Table 5 for both experiments on color FERET database, we can observe that the *Y*, *Z* ^*n*^, and *I* color components can achieve good correctness for expression, illumination, and aging experiments.

From Table 6, it can be observed that the best hybrid color spaces for both the fb subset and the dup1 subset are *YSCr*, *B* ^*n*^ *SCr*, and *Z* ^*n*^ *SCr*. Taking both the AR database and the color FERET database into consideration, we infer a result that the *Y* and *Z* ^*n*^ color components and the *YSCr*, *B* ^*n*^ *SCr*, and *Z* ^*n*^ *SCr* hybrid color spaces are very effective for our face recognition algorithm.

The comparison results for the *YSCr* hybrid color space are described in Table 7. It is shown that the proposed LCSPT-ELM outperforms in terms of computing time and recognition correctness. In this paper, the extracted feature number is very small because only 3 features for each local portion of the color component of each image are used. Naturally, training and testing time are very small. On the other hand, the feature number can be reduced by the dimension reduction techniques such as PCA and LDA. Therefore, the computational complexity can be more reduced.

**Figure 9:**
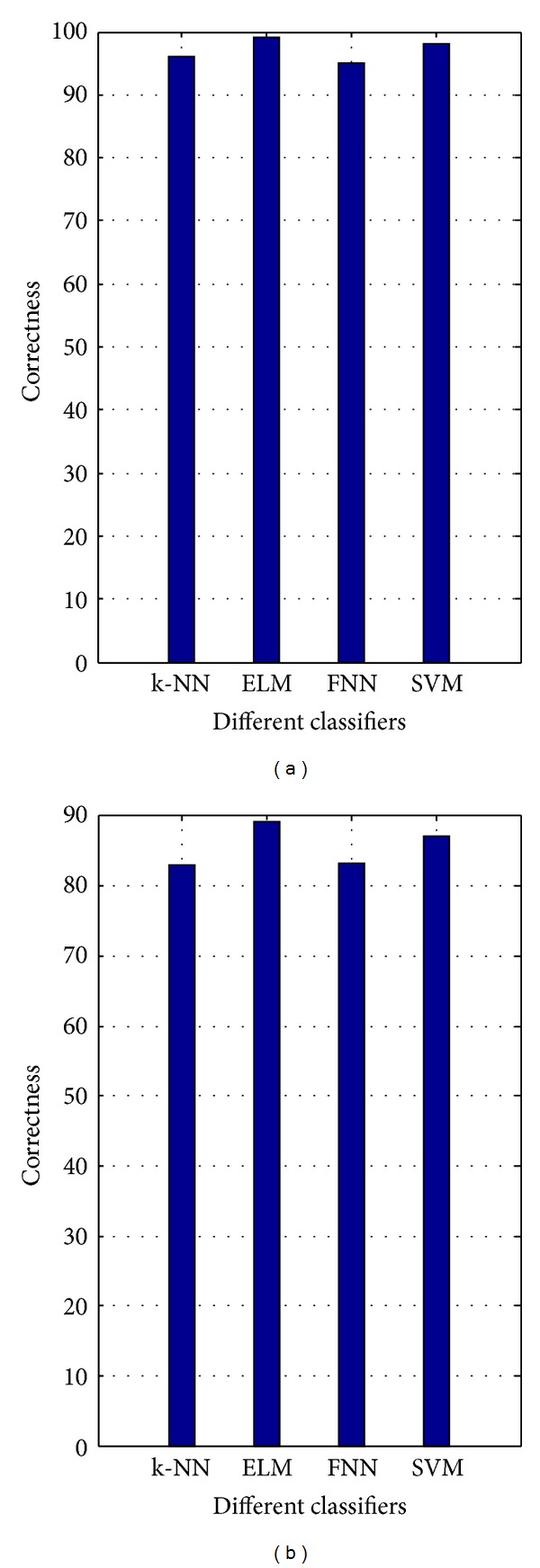
Performance comparison for k-NN, ELM, FNN, and SVM classifiers: the color FERET database: (a) fb subset and (b) dup1 subset.

Table 8 shows the average results of LCSPT face recognition algorithm using k-NN, FNN, SVM, and ELM on the *Y* color component image.  Figure 9 depicts the results of LCSPT face recognition algorithm on the *YSCr* hybrid color space. From Table 8 and Figure 9, we observe that the ELM outperforms the other classifiers.

## 7. Conclusions

This paper presents a novel face recognition algorithm by means of fusing color and local spatial information (see Supplementary Material available online at http://dx.doi.org/10.1155/2014/628494). The effectiveness of the proposed algorithm is assessed on 6 conventional color spaces, *RGB*, *HSV*, *Y* *Cb* *Cr*, *YIQ*, *XYZ*, and L*a*b*, and 6 powerful hybrid color spaces developed in the literature, *RGB*-*r* [10], *XYZ*-*n* [5], *RGB*-*n* [5], *ZRG*-*n* [5], *RIQ* [8], and *R* *Cr* *Q* [9]. In addition, 3 new hybrid color spaces are constructed in this paper. In particular, the proposed hybrid color spaces, *YSCr*, *Z* ^*n*^ *SCr*, and *B* ^*n*^ *SCr* are configured as the combination of the *Cb* and *Cr* component images of the *Y* *Cb* *Cr* color space, the *S* color component of the *HSV* color spaces, and the *Z* ^*n*^ and *B* ^*n*^ color components of the normalized *XYZ* color space. Experiments are constructed using the most challenging color FERET database and AR database.

The novelty of this paper is based on the following aspects: (i) a novel color feature extraction method is introduced by applying SPT algorithm to each color component of color face images; (ii) new hybrid color spaces are presented for improving the color face recognition performance being used together with SPT algorithm; (iii) in decision frame, the fusion of ELM classifiers is developed for fast color face recognition.

Experimental results show that SPT is an effective tool for extracting information from the color face images. The proposed LCSPT-ELM algorithm has very short training and testing time and a good recognition correctness. It is illustrated that the new hybrid color spaces of *YSCr*, *B* ^*n*^ *SCr*, and *Z* ^*n*^ *SCr* have the best performance on our algorithm. LCSPT-ELM algorithm can be used for real time face recognition applications thanks to short testing time and parameter adjusting time.

## Supplementary Material

Highlights.

## Figures and Tables

**Table 1:** Performance comparison in color component images of LCSPT-ELM face recognition algorithm: the AR database.

	ex1					ex2				
	HS	− *π*/4	0	*π*/4	*π*/2	HS	− *π*/4	0	*π*/4	*π*/2
*R*	90.97	95.13	94.44	87.50	94.44	91.96	91.07	91.36	86.30	91.36
*G*	91.66	94.44	93.05	90.97	93.05	90.77	91.66	91.66	85.71	91.07
*B*	91.66	91.66	93.13	**92.64**	94.11	93.75	93.15	93.75	**91.07**	**94.04**
*Y*	**93.75**	94.44	93.05	91.66	**94.44**	**94.04**	**93.75**	**94.64**	90.47	**94.04**
*Cb*	86.11	90.97	90.97	86.11	91.67	85.71	89.28	90.17	87.20	92.26
*Cr*	86.80	92.36	93.75	88.19	93.05	86.90	92.55	91.66	88.39	92.26
*S*	89.58	91.66	95.83	90.97	94.44	90.17	94.34	94.94	89.58	94.94
*H*	85.41	82.63	84.02	75.00	90.27	87.20	85.41	86.30	77.08	86.90
*V*	90.97	93.05	90.97	87.50	90.97	88.09	86.60	86.90	83.63	88.09
*Y*	90.97	93.05	93.75	87.50	92.36	91.36	92.26	92.85	88.69	92.26
*I*	88.89	90.27	95.13	90.27	91.66	87.20	94.04	93.75	88.09	91.36
*Q*	74.30	77.77	82.63	74.30	81.25	71.42	74.10	80.95	74.70	76.78
L*	91.66	**95.13**	93.05	90.97	93.75	91.07	94.04	93.15	89.88	93.75
a*	82.63	90.27	92.36	86.80	90.97	83.63	88.98	91.66	84.52	87.50
b*	93.62	90.68	92.15	92.15	92.15	85.71	89.28	91.36	84.82	90.47
*G* ^*n*^	90.97	92.36	93.75	88.88	94.44	91.36	92.55	92.26	88.39	92.26
*B* ^*n*^	93.05	92.36	**96.52**	91.66	93.75	92.85	93.45	**94.64**	90.47	93.15
*X* ^*n*^	92.36	94.44	94.44	90.27	**94.44**	**94.04**	**93.75**	94.04	90.77	93.75
*Y* ^*n*^	87.50	89.58	90.97	86.80	88.19	89.28	88.98	91.66	86.90	89.88
*Z* ^*n*^	90.97	92.36	**96.52**	92.36	**94.44**	93.45	93.15	94.04	90.47	**94.04**
*X*	89.58	94.44	93.75	86.80	90.97	90.47	92.55	92.26	88.69	92.85
*Z*	92.36	93.05	95.13	88.19	93.75	92.85	92.55	92.26	88.09	93.45
*Gr*	85.41	92.36	94.44	87.50	92.36	82.73	93.75	90.17	86.30	89.28
*Br*	89.58	92.36	92.36	88.88	94.44	87.79	91.36	93.45	87.79	94.94

Bold values mean the color components with correctness over 90% for all subbands and the subbands with the highest correctness.

**Table 2:** Performance comparison in different hybrid color spaces of LCSPT-ELM face recognition algorithm: the AR database.

	ex1					ex2				
	HS	−*π*/4	0	*π*/4	*π*/2	HS	−*π*/4	0	*π*/4	*π*/2
*RG* *B*	97.72	98.41	97.02	96.33	97.02	96.78	97.08	96.28	94.40	97.08
*HS* *V*	95.25	96.61	94.72	95.64	94.69	93.51	94.58	97.58	95.59	93.98
*Y* *Cb* *Cr*	95.63	98.41	97.71	94.94	98.41	95.88	98.26	97.07	94.39	97.07
*YI* *Q*	96.33	97.02	97.72	95.64	96.33	95.88	97.07	97.67	95.88	96.78
*XY* *Z*	94.24	95.63	96.33	90.77	96.33	93.50	94.99	94.39	90.23	94.99
L*a*b*	94.02	99.01	97.02	97.41	98.41	96.78	98.16	97.37	95.98	98.08
*RG* *B*-*n* [5]	93.75	96.82	97.71	95.64	95.69	94.99	97.85	97.37	94.99	97.07
*XY* *Z*-*n* [5]	93.55	97.02	97.72	95.64	97.72	94.99	95.59	95.59	92.91	95.29
*RG* *B*-*r* [10]	94.25	97.02	97.72	95.64	97.72	94.69	98.56	97.37	94.10	97.97
*RI* *Q* [8]	94.94	97.71	97.71	96.33	98.41	94.39	96.18	95.88	93.80	95.88
*R* *Cr* *Q* [9]	96.33	97.72	97.72	95.64	98.41	94.69	96.28	96.18	93.80	96.18
*Z* *RG*-*n* [5]	97.02	97.72	97.02	94.94	97.02	96.18	96.48	97.37	94.10	98.26
*YS* *Cr*	96.33	99.11	**99.80**	97.72	99.11	96.78	98.86	96.26	96.78	**99.45**
*B* ^*n*^ *S* *Cr*	95.64	99.11	**99.80**	97.02	99.11	97.07	**99.16**	98.56	96.78	**99.16**
*Z* ^*n*^ *S* *Cr*	95.64	99.11	**99.80**	98.41	99.11	96.18	**99.16**	98.56	96.78	**99.16**

Bold values specify the hybrid color spaces and their subbands with the highest correctness.

**Table 3:** Comparison of training time, testing time, and correctness of LCVBP, LBD, and LCSPT-ELM: the AR database.

	ex1			ex2		
	Training time (s)	Testing time (s)	Correctness rate (%)	Training time (s)	Testing time (s)	Correctness rate (%)
LCVBP [39]	5850.12	5320.45	98.01	10350	7750	97.56
LBP [38]	3826.01	—	98.23	3795.91	—	98.12
LCSPT-ELM	544.86	360.27	99.80	602.52	358.08	99.16

**Table 4:** Comparison of parameter adjusting time and testing time of k-NN, FNN, SVM, and ELM: the AR database.

	ex1			ex2		
	Parameter adjusting time (s)	Testing time (s)	Best parameters	Parameter adjusting time (s)	Testing time (s)	Best parameters
k-NN	4.3	0.12	*k* = 2	10.27	0.38	*k* = 2
SVM	119	0.28	(2^1^, 2^−10^)	508.12	1.157	(2^1^, 2^−10^)
ELM	12.79	0.034	*N* = 104	82.54	0.080	*N* = 122
FNN	1875	0.028	*N* = 44	11494	0.032	*N* = 54

**Table 5:** Performance comparison in the color component images of LCSPT-ELM face recognition algorithm: the color FERET database.

	fb set					dup1 set				
	HS	−*π*/4	0	*π*/4	*π*/2	HS	−*π*/4	0	*π*/4	*π*/2
*R*	91.19	93.15	91.68	89.72	91.68	73.35	71.88	74.82	72.37	73.35
*G*	91.19	91.68	94.13	92.66	93.64	**78.25**	72.37	77.27	69.43	73.84
*B*	92.66	92.66	94.13	93.64	**95.11**	70.90	71.39	71.39	68.45	74.82
*Y*	91.70	92.66	**95.64**	91.19	93.64	72.86	70.90	**84.39**	**75.39**	74.82
*Cb*	94.62	92.17	94.62	**95.11**	94.62	69.92	64.03	73.35	61.09	73.84
*Cr*	93.64	93.15	93.15	89.72	92.66	75.80	75.31	79.72	67.47	77.27
*S*	90.70	**95.60**	95.11	94.13	96.09	70.41	69.43	70.90	72.86	77.27
*H*	92.66	91.19	91.68	87.27	93.15	69.43	62.56	69.92	55.70	65.50
*V*	87.76	92.66	92.17	90.21	91.68	69.43	73.84	74.82	71.88	74.82
*Y*	89.72	92.66	94.62	91.19	94.13	71.39	72.37	76.29	74.82	76.78
*I*	92.66	93.64	**95.64**	93.15	94.62	76.29	**77.76**	80.21	72.37	82.17
*Q*	73.05	74.03	76.98	75.50	76.98	49.82	53.25	56.19	52.76	55.21
L*	92.17	92.17	93.64	91.68	93.64	73.35	71.88	70.90	70.41	73.35
a*	93.64	91.68	90.21	90.70	93.64	74.82	69.43	76.78	63.05	76.78
b*	94.62	91.68	93.15	93.15	93.15	66.49	65.50	71.88	66.49	72.86
*G* ^*n*^	93.15	92.66	93.64	91.19	94.13	70.41	69.43	74.33	66.49	71.88
*B* ^*n*^	91.70	92.66	94.13	92.17	93.15	68.94	69.92	70.41	68.94	75.31
*X* ^*n*^	91.68	93.15	93.64	91.19	92.17	73.84	70.90	69.92	69.43	74.82
*Y* ^*n*^	91.68	89.23	89.23	89.72	91.19	71.39	65.01	70.41	60.60	69.92
*Z* ^*n*^	92.17	91.68	94.62	92.17	**95.11**	69.92	68.45	73.35	68.45	**83.29**
*X*	90.21	93.64	**95.64**	91.19	92.66	69.43	72.37	76.78	75.37	76.78
*Z*	88.74	92.66	94.13	90.21	93.15	66.00	67.47	73.84	69.92	73.84
*Gr*	92.66	92.17	92.66	90.70	94.13	74.82	72.86	76.78	65.50	77.27
*Br*	**96.58**	95.11	94.13	94.13	93.64	72.85	74.33	75.80	71.88	77.76

Bold values specify the color components and their subbands with high correctness.

**Table 6:** Performance comparison in different hybrid color spaces of LCSPT-ELM face recognition algorithm: the color FERET database.

	fb set					dup1 set				
	HS	−*π*/4	0	*π*/4	*π*/2	HS	−*π*/4	0	*π*/4	*π*/2
*RG* *B*	97.79	97.790	97.30	96.81	96.81	76.86	73.92	74.41	73.43	75.88
*HS* *V*	90.42	96.850	97.71	93.34	95.92	80.32	80.34	80.24	78.11	85.17
*Y* *Cb* *Cr*	99.26	97.30	98.77	98.28	98.77	82.74	84.70	84.70	81.27	84.21
*YI* *Q*	96.32	97.30	98.77	96.32	97.79	75.88	77.35	82.25	76.86	80.29
*XY* *Z*	91.42	94.85	96.81	93.38	96.32	70.00	72.94	76.86	75.88	78.33
L*a*b*	92.12	97.88	97.30	97.17	98.81	80.74	83.23	83.41	82.43	84.84
*RG* *B*-*n* [5]	93.89	94.85	97.83	93.87	95.32	80.68	84.70	83.23	80.80	79.25
*XY* *Z*-*n* [5]	92.89	94.85	96.81	93.87	96.32	74.90	73.43	75.88	75.88	79.31
*RG* *B*-*r* [10]	99.70	96.81	97.30	95.83	97.79	80.78	84.70	84.21	79.80	82.25
*RI* *Q* [8]	95.83	96.81	98.28	96.81	98.28	75.39	80.29	82.74	79.31	79.80
*R* *Cr* *Q* [9]	98.28	97.30	97.79	97.79	96.81	76.37	79.80	81.76	79.31	81.76
*Z* *RG*-*n* [5]	94.36	94.85	95.83	93.87	96.32	75.88	75.88	77.35	74.90	77.84
*YS* *Cr*	96.81	98.28	98.77	98.28	**99.75**	85.19	84.70	84.70	83.23	**89.11**
*B* ^*n*^ *S* *Cr*	97.79	98.28	98.77	99.26	**99.75**	82.74	84.21	83.72	81.27	**89.11**
*Z* ^*n*^ *S* *Cr*	97.79	98.28	98.77	99.26	**99.75**	83.72	83.72	85.19	82.74	**89.11**

Bold values specify the hybrid color spaces and their subbands with the highest correctness.

**Table 7:** Comparison of training time, testing time, and correctness of LCVBP, LBD, and LCSPT-ELM: the color FERET database.

	fa set			dup1 set		
	Training time (s)	Testing time (s)	Correctness rate (%)	Training time (s)	Testing time (s)	Correctness rate (%)
LCVBP [39]	8021.77	7406.13	97.12	7676.57	8338.85	82.43
LBP [38]	3700.91	—	98.03	3091.71	—	88.49
LCSPT-ELM	544.86	360	99.75	602.52	358	89.11

**Table 8:** Comparison of parameter adjusting time and testing time of k-NN, FNN, SVM, and ELM: the color FERET database.

	fb set			dup1 set		
	Parameter adjusting time (s)	Testing time (s)	Best parameters	Parameter adjusting time (s)	Testing time (s)	Best parameters
k-NN	16.98	0.1512	*k* = 2	12.37	0.15	*k* = 2
SVM	99.12	1.23	(2^−2^, 2^−10^)	91.32	1.12	(2^−2^, 2^−10^)
ELM	16.38	0.06	*N* = 120	15.28	0.032	*N* = 114
FNN	2325.80	0.02	*N* = 52	2313.62	0.02	*N* = 58
