# Supplementary material for: Color Face Recognition Based on Steerable Pyramid Transform and Extreme Learning Machines
Source: ScientificWorldJournal. 2014 Jan 16;2014:628494. doi: 10.1155/2014/628494 (PMC3914600; doi:10.1155/2014/628494)
Supplement: Supplementary file 1 — Highlights. [file 628494.f1.docx]

**Highlights**

This paper presents a novel color face recognition algorithm by means of fusing color and local information.

Multi-orientation and multi-scale information relating to the color face features are extracted by applying Steerable Pyramid Transform (SPT) to the local face regions.

All features are fused according to decision fusion frame and the combinations of Extreme Learning Machine (ELM) classifiers are applied for color face recognition with fast and high correctness.

The experiments on the AR database and the Color FERET database show that the proposed Local Color Steerable Pyramid Transform (LCSPT) face recognition algorithm improves face recognition performance seriously on the novel hybrid color spaces such as YSCr, Z^n^SCr, and B^n^SCr.

The proposed method achieves faster recognition compared with state-of-the-art studies.
